# TOPSY: A picture description dataset to examine speech, language and communication in untreated first episode psychosis

**DOI:** 10.1016/j.dib.2026.112788

**Published:** 2026-04-24

**Authors:** Sharan Bhullar, Chaimaa El Mouslih, Michael Mackinley, Julie Richard, Lena Palaniyappan

**Affiliations:** a London Health Sciences Centre Research Institute (LHSCRI), London, Ontario, Canada; b Department of Psychology, McGill University, Montreal, Quebec, Canada; c Douglas Mental Health University Institute, McGill University, Montreal, Quebec, Canada; d Department of Psychiatry, Western University, London, Ontario, Canada

**Keywords:** Schizophrenia, Natural language processing, Voice, Digital health

## Abstract

Recordings of human behaviour provide a rich source of data to detect features of conditions such as psychosis. Advanced technologies can now detect subtle speech aberrations that often go unnoticed by clinicians and family members. This is particularly important given that behavioral data, especially from untreated samples, are scarce. Speech is a crucial verbal behavioral data point for understanding psychosis, but the lack of comprehensive datasets has been a major limitation in research. Furthermore, long-term antipsychotic treatment can significantly affect speech, impacting both its tone and content. This makes it critical to collect longitudinal data to understand these effects. Tracking Outcomes of Psychosis (TOPSY) study aims to address these gaps by providing a rich dataset that includes not only speech data collected using a semi-structured picture description protocol but also a range of other valuable measures based on a validated Thought and Language Index. The TOPSY dataset offers naturalistic speech samples from individuals in an early-stage psychosis treatment program (<2 median days of antipsychotic treatment) and demographically matched healthy controls. This data is complemented by a comprehensive neurobiological and clinical battery, including: MRS (Magnetic Resonance Spectroscopy), Structural MRI, T1 structural data with myelin mapping, DTI (Diffusion Tensor Imaging), Resting fMRI (functional Magnetic Resonance Imaging). In addition to the neuroimaging data, we have collected detailed clinical and cognitive information, including TLI scores (Thought and Language Index) verbal fluency, modified DSST, and PANSS-8 item scores. The protocol and clinical assessments were repeated after a 12-month interval to assess the longitudinal stability of speech, symptom burden, and functional status. Transcripts were extracted from conversations lasting between 3 and 5 min, allowing for in-depth analysis of acoustic, semantic, syntactic, and pragmatic measures related to psychosis. We expect this dataset to be invaluable for future investigations into the clinical utility of speech measures for assessing thought disorder and other psychosis-related symptoms.

Specifications Table

**Table:** 

Subject	Health Sciences, Medical Sciences & Pharmacology
Specific subject area	Clinical linguistics, AI, psychopathology in psychosis.
Type of data	Audio files (.mp3 format) Transcription (.CLAN format) Table (.csv format)
Data collection	The data were collected using the following methods: - Standardized questionnaires capturing clinical (8 items of PANSS) and cognitive data. - A semi-structured protocol to elicit speech during a picture description task (3 pictures), with the full interview recorded using an Olympus or SONY voice recorder. - Transcriptions generated using the Batchalign Python program in the CHAT/CLAN system through PsychosisBank. The data collection period spanned from April 2017 to October 2025. Participants were interviewed in a quiet room with minimal distractions. A subset of participants underwent a repeat assessment one year later. The dataset supporting these elements is available through PsychosisBank [1].
Data source location	The data were collected in London, Ontario, and the curated dataset is stored in PsychosisBank.
Data accessibility	Repository name: PsychosisBank Data identification number: *(or DOI or persistent identifier)* Direct URL to data: https://sla.talkbank.org/TBB/psychosis/English/Palaniyappan/ Instructions to Access Data: Access to the Data is granted by the DISCOURSE Consortium Data Access Committee to approved researchers (No access will be granted for commercial purposes). For complete instructions on becoming a Psychosisbank member please see: https://talkbank.org/psychosis/membership.html
Related research article	Dalal TC, Liang L, Silva AM, Mackinley M, Voppel A, Palaniyappan L. Speech based natural language profile before, during and after the onset of psychosis: A cluster analysis. Acta Psychiatrica Scandinavica. 2025 Mar;151(3),332–347.

## 1 Value of the Data

• TOPSY includes a well-characterized clinical sample with clinical high-risk (CHR), first-episode psychosis (FEP), and established schizophrenia on long-acting injectables (SZ) in addition to a healthy control group. This spectrum allows comparative work across illness stages.
• Stringent recruitment and diagnostic validity: FEP patients were recruited within one week of referral to an early intervention team and speech was recorded with <14 days of lifetime antipsychotic exposure (most <3 days). This allows the study of negative thought disorder without confounds from long-term antipsychotic medications.
• Multidomain clinical assessment with PANSS-8, TLI, CDSS, CAST (cannabis) and SOFAS provides thorough assessments of positive, negative, disorganization, cannabis use, mood and global functioning domains.
• Speech and language recordings: Semi-standardized elicitation using picture description/tasks aligned with TLI, providing three minutes of clinically relevant, elicited (not passive) speech per subject, transcribed and cleaned. These datasets are highly valuable, as well‐annotated psychosis speech‐language datasets are rare. The possibility of combining these data with MRI neuroimaging data provides an opportunity to study the neurobiology of language in psychosis.

## 2 Background

Language disturbance is a hallmark of psychotic disorders, predicting illness onset [2,3], relapses [4,5], and impaired quality of life [6]. Advances in computational linguistics now allow objective quantification of these disturbances, but model development and replication depend on shared datasets [7].

The TOPSY dataset integrates standardized clinical assessments with transcribed speech samples from 258 sessions from 199 participants: healthy controls, individuals at clinical high risk (CHR), first-episode psychosis (FEP) patients assessed within one week of referral with minimal antipsychotic exposure, and clinically stable patients with schizophrenia (SZ). Speech was elicited using picture-description tasks, recorded, transcribed, and processed to support validated natural language processing tools capturing lexical, syntactic, and semantic-cohesion features. Picture-description paradigms are particularly sensitive to psychosis-related speech changes compared to other elicitation methods (e.g., word list generation) [8]. Follow-up assessments (6–12 months after treatment onset) provide additional information on longitudinal trajectories [see 9,10].

By enabling analysis of linguistic variables alongside detailed clinical phenotyping across illness stages, TOPSY advances four areas: (1) characterizing thought-disorder phenomenology, (2) developing speech-based predictive and monitoring tools, (3) deep phenotyping in early and established psychosis, and (4) identifying linguistic markers linked to functioning and prognosis. This expert-labelled dataset addresses a key gap in computational psychiatry and supports collaborative discovery.

## 3 Data Description

The files included in this dataset are:

1. The dataset consists of four integrated modalities—audio recordings, corresponding transcripts, clinical and demographic measures, and high-resolution neuroimaging data—each linked through a unique participant identifier. Together, these components capture comprehensive information on speech production, linguistic output, symptom profiles, functioning, and brain structure and function across baseline and one-year follow-up assessments, enabling multimodal analyses of language, clinical features, and neurobiological markers. This dataset is hosted through PsychosisBank [1].
2. Audio files in .mp3 format include 1-minute descriptions of three pictures from the Thematic Apperception Test [TAT] [11] for a total of three minutes of speech.
3. Transcription of the audio files are provided in .clan format for picture description tasks [three TAT pictures] from both baseline and one-year follow-up.
4. Clinical and Demographic Data: Socio-demographic data of patients and healthy controls include consensus diagnosis, gender, hospitalization status, education, employment, education/training, job, language, ethnicity, and immigration status. The study also collected clinical data of self-endorsed substance use, psychotropic drugs, symptom severity in schizophrenia, occupational and educational functioning data, and occurrence of relapses over 1 year. Additionally, the dataset includes scores for verbal fluency and modified digit symbol substitution.
5. Imaging Data: Participants underwent a 75-minute 7T MRI protocol that included structural imaging, anterior cingulate MRS [12], myelin-sensitive imaging [13], resting-state fMRI [14], and diffusion tensor imaging (DTI) [15].

### Sociodemographic Descriptions

**Table:** 

Sociodemographic Factor	Classifications
Patient Category	Healthy Control, Patient
Consensus Diagnosis	Schizophrenia, Schizoaffective, Bipolar with psychotic features, Psychosis NOS
Sex	Female, Male
Education	≤Gr9, ≤Gr 12, HS, College/Vocational, BA/BSC, MA/MSC/PhD
Parental Socioeconomic status (SES)	Higher occupations/Professional, Intermediate/Management, Self-employed/Own account, Lower supervisory/technical, Semi routine and routine
NEET status	Binary marker of vocational inactivity defined as “not in employment, education, or training”.

### Cognitive and Clinical Scores

**Table:** 

Metric	Definition
AUDIT-C scores	Measures of alcohol use
Smoking Index	(Number of cigarettes/day x years of tobacco use)/20
Cannabis Abuse Screening Test (CAST) scores	Self-reported screening measure of cannabis use
Substance Use Questionnaire (SUQ)	Self-reported use of alcohol and other recreational substances
Positive and Negative Syndrome Scale (PANSS) – 8 Item Version	Scale to assess psychosis related symptom burden
Calgary Depression Scale	Scale to assess depressive burden in individuals with schizophrenia
Young Mania Rating Scale	Measures the severity of manic symptoms
Social and Occupational Functioning Assessment Scale (SOFAS)	Index for the assessment of social and occupational functioning
Category Fluency Score	Test of semantic verbal fluency
Modified Digit Symbol Substitution Test	Assessment of cognition for processing speed
Thought and Language Index	Instrument for assessing formal thought disorder (metrics of both impoverishment and disorganization) based on a picture description task.

## 4 Experimental Design, Materials and Methods

### 4.1 Participants

199 participants were recruited from the Prevention and Early Intervention Program for Psychosis in London, Ontario, along with 58 healthy controls. The participants were 16–50 years old, met the operational criteria for a psychosis disorder as per the Diagnostic and Statistical Manual of Mental Disorders, Fifth Edition (DSM-5), and were diagnosed with a psychotic disorder by a psychiatrist.

Those with psychosis secondary to a substance use disorder or with an active dependence pattern in the past year, or a known neurological disorder impacting speech output (e.g., apraxia) were excluded.

**Table:** 

Variable	First Episode Patients: n = 109 sessions	Chronic: 49	Clinical High-Risk: 25	Healthy Controls: 75
Sex (M/F)	89 /20	38/11	7/18	51/24
Age at baseline [m (SD)]	22.41 (4.39) years	29.95 (6.55) years	22.16 (4.18) years	23.13 (4.72) years
NS-SEC [m (SD)]	3.59 (1.32)	3.51 (1.16)	3.88 (0.83)	2.89 (1.31)
Education	12.72 (1.70)	12.94 (2.19)	13.00 (1.84)	14.45 (2.12)
PANSS-8 item total	21.71 (8.34)	13.43 (5.09)	8.72 (1.74)	8.00 (0.00)

*Note:* NSSEC: Parental socioeconomic data based on National Statistics Socio-economic Classification of Rose and Pevalin; PANSS-8: 8 items of Positive and Negative Symptoms Scale. Variables are summarised based on total number of sessions based on available data. Each visit (baseline, 6-12 months, 12-18 months) is counted as a session.

### 4.2 Experimental Procedure

Participants had one or more visits: baseline visit, and a 6-12 months follow-up. Participants underwent a complete clinical, cognitive, and speech sample collection at each assessment in addition to the 75-minute 7T MRI brain scan.

### 4.3 Baseline Visit

*Patient Interview*

Following the completion of all consent procedures, patients were assessed using the Social and Occupational Functioning Assessment Scale (SOFAS), Substance Use Questionnaire (SUQ), Positive and Negative Syndrome Scale- 8 Item Version (PANSS-8), Calgary Depression Scale (CDS), Young Mania Rating Scale (YMRS), Category Fluency Test, and Digit Symbol Substitution Test were administered.

*THOUGHT and LANGUAGE INDEX (TLI) Protocol*

As one component of the seven discourse tasks, participants completed speech tasks drawn from the Thought and Language Index – Short Form [16,17] following the clinical and cognitive questionnaires. The TLI is a structured tool used to assess formal thought disorder through analysis of spontaneous speech. General information about the DISCOURSE project is available at https://discourseinpsychosis.org.

If participants paused for >10 s or did not initiate speech for 30 s, neutral prompts such as “Can you tell me more?” or “Anything else?” were used to encourage verbal output. The task involves asking participants to describe three pictures taken from the Thematic Apperception Test, speaking for at least 30 s and up to 2 min per image. This approach elicits narrative responses that provide referents containing multiple descriptive components [16].

The narratives are then scored across multiple domains of thought and language disturbance, including poverty of speech, tangentiality, illogicality, and peculiar word usage, according to the TLI short-form criteria [16,10]. Detailed training and scoring guidelines are available from the original TLI authors.

### 4.4 Data collection

The speech samples were recorded with an Olympus or SONY voice recorder. The recorder was positioned close enough to both staff and participant so that the discourse could be captured from both speakers.

### 4.5 Data processing

Transcriptions were automatically generated with Batchalign, a Python program that creates CHAT transcripts from recorded audio files [18]. The generated transcripts were then manually verified by researchers with the original audio recordings using the CLAN program [18]. Original audio files and the verified transcripts were then uploaded to the PsychosisBank servers.

## Limitations

While the data provides many avenues for investigation, we report several limitations. As recruitment of patients was coducted largely within a first-episode psychosis clinic, our sample is likely biased to patients who are actively engaged in treatment and may not reflect the full spectrum speech in psychosis. A limitation of the current dataset is the imbalance in sex distribution within the first-episode psychosis (FEP) group (89 male, 20 female participant sessions). Given established sex differences in psychosis phenomenology and speech patterns, this imbalance should be considered when interpreting findings and may affect the generalizability of results.

## Ethics Statement

Written informed consent was obtained from all patients/participants prior to participation in the study. The Research Ethics Board at Western University approved all study procedures.

## CRediT Author Statement

**Sharan Bhullar:** Validation and visualisation, writing - original draft, & editing; **Chaimaa El Mouslih :** Validation and visualisation; **Michael Mackinley:** Investigation, Formal analysis, data curation, writing - review, & editing, project administration; **Julie Richard :** Project administration; **Lena Palaniyappan:** Conceptualization, methodology, software, writing - review & editing, supervision, project administration.

## Declaration of Competing Interest

LP reports personal fees for serving as chief editor from the Canadian Medical Association Journals, speaker honorarium from Janssen Canada and Otsuka Canada, SPMM Course Limited, UK; book royalties from Oxford University Press; investigator-initiated educational grants from Otsuka Canada outside the submitted work, in the last 5 years.

## Data Availability

PsychosisBankTOPSY: A Picture Description Dataset to Examine Speech, Language, and Communication in Untreated First Episode Psychosis (Original data).

## References

[1] Palaniyappan L., Bhullar S., El Mouslih C., Mackinley M., Richard J., the TOPSY Research Team (2025). *TOPSY: transcribed picture-description dataset across the psychosis spectrum* [Data set]. PsychosisBank (DISCOURSE Consortium.

[2] Corcoran C.M., Carrillo F., Fernández-Slezak D., Bedi G., Klim C., Javitt D.C., Bearden C.E., Cecchi G.A. (2018). Prediction of psychosis across protocols and risk cohorts using automated language analysis. World Psychiatry.

[3] Mota N.B., Weissheimer J., Finger I., Ribeiro M., Malcorra B., Hübner L. (2023). Speech as a graph: developmental perspectives on the organization of Spoken Language. Biol. Psychiatry Cogn. Neurosci. NeuroimAging.

[4] Dalal T.C., Liang L., Silva A.M., Mackinley M., Voppel A., Palaniyappan L. (2025). Speech based natural language profile before, during and after the onset of psychosis: a cluster analysis. Acta Psychiatr. Scand..

[5] Zaher F., Diallo M., Achim A.M., Joober R., Roy M.A., Demers M.F., Subramanian P., Lavigne K.M., Lepage M., Gonzalez D., Zeljkovic I., Davis K., Mackinley M., Sabesan P., Lal S., Voppel A., Palaniyappan L. (2024). Speech markers to predict and prevent recurrent episodes of psychosis: a narrative overview and emerging opportunities. Schizophr. Res..

[6] Mackinley M., Limongi R., Silva A.M., Richard J., Subramanian P., Ganjavi H., Palaniyappan L. (2023). More than words: speech production in first-episode psychosis predicts later social and vocational functioning. Front. Psychiatry.

[7] Corona Hernández H., Corcoran C., Achim A.M., de Boer J.N., Boerma T., Brederoo S.G., Cecchi G.A., Ciampelli S., Elvevåg B., Fusaroli R., Giordano S., Hauglid M., van Hessen A., Hinzen W., Homan P., de Kloet S.F., Koops S., Kuperberg G.R., Maheshwari K., Mota N.B., Palaniyappan L. (2023). Natural language processing markers for psychosis and other psychiatric disorders: emerging themes and research agenda from a cross-linguistic workshop. Schizophr. Bull..

[8] Morgan S.E., Diederen K., Vértes P.E., Ip S.H.Y., Wang B., Thompson B., Demjaha A., De Micheli A., Oliver D., Liakata M., Fusar-Poli P., Spencer T.J., McGuire P. (2021). Natural Language processing markers in first episode psychosis and people at clinical high-risk. Transl. Psychiatry.

[9] Alonso-Sánchez M.F., Ford S.D., MacKinley M., Silva A., Limongi R., Palaniyappan L. (2022). Progressive changes in descriptive discourse in First Episode Schizophrenia: a longitudinal computational semantics study. Schizophrenia.

[10] Sommer I.E., Bearden C.E., van der Meer L., Kahn R.S. (2010). The Thought and Language Index: a short form for assessing thought and language in schizophrenia. Schizophr. Res..

[11] Murray H.A. (1949).

[12] Wang Y.L., Sharpe V., Mackinley M., Kuperberg G.R., Supekar K., Theberge J., Palaniyappan L. (2025). Glutamate, contextual insensitivity and disorganized speech in first-episode schizophrenia: a 7-tesla magnetic resonance spectroscopy study. *Biol. Psychiatry Glob. Open. Sci.*,.

[13] Park M.T.M., Jeon P., French L., Dempster K., Chakravarty M.M., MacKinley M., Richard J., Khan A.R., Théberge J., Palaniyappan L. (2022). Microstructural imaging and transcriptomics of the basal forebrain in first-episode psychosis. Transl. Psychiatry.

[14] Limongi R., Silva A.M., Mackinley M., Ford S.D., Palaniyappan L. (2023). Active inference, epistemic value, and uncertainty in conceptual disorganization in first-episode schizophrenia. Schizophr. Bull..

[15] Van Dyken P.C., Yang K., Faria A.V., Sawa A., MacKinley M., Khan A.R., Palaniyappan L. (2025). Stable white matter structure in the first three years after psychosis onset. Biol. Psychiatry Glob. Open. Sci..

[16] Liddle P.F., Ngan E.T.C., Caissie S.L., Anderson C.M., Bates A.T., Quested D.J., White R., Weg R. (2002). Thought and Language Index: an instrument for assessing thought and language in schizophrenia. Br. J. Psychiatry.

[17] Silva A.M., Limongi R., MacKinley M., Ford S.D., Alonso-Sánchez M.F., Palaniyappan L. (2023). Syntactic complexity of spoken language in the diagnosis of schizophrenia: a probabilistic Bayes network model. Schizophr. Res..

[18] MacWhinney B. (2025). Understanding language through TalkBank. Curr. Dir. Psychol. Sci..

